# Glycosylated-Chitosan Derivatives: A Systematic Review

**DOI:** 10.3390/molecules25071534

**Published:** 2020-03-27

**Authors:** Pasquale Sacco, Michela Cok, Francesca Scognamiglio, Chiara Pizzolitto, Federica Vecchies, Andrea Marfoglia, Eleonora Marsich, Ivan Donati

**Affiliations:** 1 Department of Life Sciences, University of Trieste, Via Licio Giorgieri 5, I-34127 Trieste, Italy; psacco@units.it (P.S.); mcok@units.it (M.C.); fvecchies@units.it (F.V.); ANDREA.MARFOGLIA@studenti.units.it (A.M.); 2 Department of Medicine, Surgery and Health Sciences, University of Trieste, Piazza dell’Ospitale 1, I-34129 Trieste, Italy; fscognamiglio@units.it (F.S.); CHIARA.PIZZOLITTO@phd.units.it (C.P.); emarsich@units.it (E.M.)

**Keywords:** chitosan, glycosylated-chitosan derivatives, chemical synthesis, physical-chemical properties, drug/gene delivery applications, tissue engineering applications

## Abstract

Chitosan derivatives, and more specifically, glycosylated derivatives, are nowadays attracting much attention within the scientific community due to the fact that this set of engineered polysaccharides finds application in different sectors, spanning from food to the biomedical field. Overcoming chitosan (physical) limitations or grafting biological relevant molecules, to mention a few, represent two cardinal strategies to modify parent biopolymer; thereby, synthetizing high added value polysaccharides. The present review is focused on the introduction of oligosaccharide side chains on the backbone of chitosan. The synthetic aspects and the effect on physical-chemical properties of such modifications are discussed. Finally, examples of potential applications in biomaterials design and drug delivery of these novel modified chitosans are disclosed.

## 1. Chitosan and Its Derivatives

Chitosan is a polysaccharide that is commercially derived from chitin—the second most abundant polysaccharide on earth. Chitin is the main component of the exoskeleton of Arthropoda, although cell walls of fungi, such as zygomycetes, contain it in small amounts [1]. The term chitosan refers to a family of polysaccharides composed of β-1→4 linked D-glucosamine units (deacetylated units, D) interspersed by residual *N*-acetyl-D-Glucosamines (acetylated units, A). The two monomers are randomly distributed along the polymer chain, although a limited block-wise structure has been reported for chemically re-acetylated chitosan samples [2].

Due to its good biocompatibility, chitosan has been proposed for several applications, spanning from biomaterials and tissue engineering to antibacterial, antifungal, antitumor, and antioxidant agent [1]. Several chemical modifications of chitosan have been proposed to endow the polysaccharide with specific properties. In fact, modified chitosan samples can act as stimuli sensitive materials (pH-, thermo-, or light-sensitive) upon phosphorylation [3], quaternarization [4], carboxylation [5], sulfonation [6], *N*-alkylation, and acylation [7].

Given the residual degree of acetylation, which ranges typically between 5% and 40% for commercial polymers, chitosan is soluble only at acidic pH. This aspect limits sensibly the application of this polysaccharide in the biomaterials field, as such, and in combination with other biologically relevant polysaccharides (e.g., alginate and hyaluronan, to name a few). To this end, chemical modifications altering the aqueous solubility of chitosan are highly sought. Among these, glycosylation represents an interesting approach also in view of the biological relevance displayed by oligosaccharides [8,9].

## 2. Synthesis of Glycosylated-Chitosan Derivatives

Aiming to increase chitosan aqueous solubility, the introduction of oligosaccharides on the chitosan backbone, exploiting the presence of primary amino groups, is of crucial interest. Particularly, mono- and di-saccharides, due to their availability and low cost, have been extensively used to obtain soluble branched chitosans. It is possible to summarize the most popular chemical modifications into three main approaches: (*i*) reductive *N*-alkylation, (*ii*) amide bond formation and (*iii*) Maillard reaction. These reactions allow obtaining a wide range of substitution degrees, depending not only on the operating conditions, but also on the type of saccharide employed. At a glance, focusing on the *N*-alkylation reaction in 1984, Yalpani reported a series of mono-, di-, tri-, and polysaccharides conjugated to the free amino-function at C2 of chitosan [10]. In this investigation, the authors exposed a range of substitution degrees from 1% to 97% of free amino groups quantified through elemental analysis. Sashiwa and Shigemasa increased the library of glycosylated chitosans with additional *N*-alkylated derivatives [11]. In particular, they obtained a range of substitution degrees, determined by ^1^H-NMR, from 37% to 100% for monosaccharide-modified chitosan and from 7% to 74% for disaccharide-modified chitosan. Concerning the amide bond formation, the degree of condensation between the free amino groups of chitosan, and the acidic group introduced in the saccharide, turned out to be lower than the other reactions described. In particular, the reaction between chitosan and lactobionic acid has been investigated by Il’ina et al. and different degrees of substitution (from 8% to 23%) have been reached [12]. On the other hand, the Maillard reaction has been exploited, in particular for the conjugation of chitosan with glucose. Glucose-modified chitosan has been largely characterized by Chung et al. and subsequently by Gullón et al. [13,14] In particular, the latter group focused their research on the optimization of the reactive conditions obtaining a Chit-Glc derivative with a degree of substitution of 64.76 ± 4.40%.

### 2.1. Reductive N-Alkylation

**Scheme 1:**
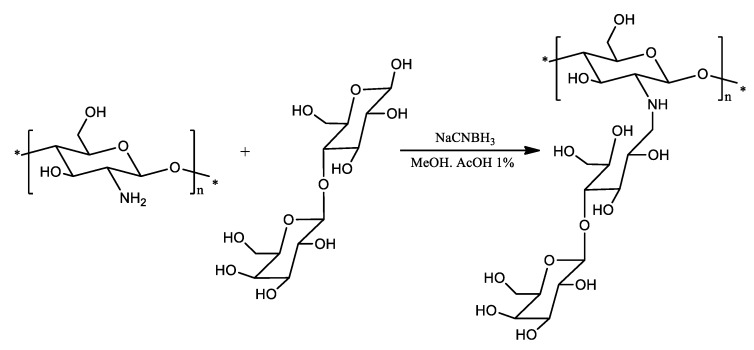
Reductive *N*-alkylation of chitosan using lactose as oligosaccharide. Redrawn from [15].

The use of reductive *N*-alkylation for the covalent attachment of oligosaccharides to the primary amino functions of chitosan is a simple and versatile procedure. In 1984, Yalpani and Hall reported various *N*-alkylated chitosan derivatives with various saccharides obtained by means of this approach [10]. Later, a lactose-modified chitosan obtained in a similar way (Scheme 1) arose great interest, in terms of biological applications, due to its good biocompatibility and bioactivity [15].

However, the synthesis of *N*-alkylated chitosan as originally devised presents two critical aspects: the use of NaCNBH_3_ (known for its toxicity) and a long dialysis process required for product purification. For these reasons, it has been necessary to improve the operative conditions, the purification and, consequently, the reaction yield. The NaCNBH_3_ has been successfully replaced by a non-toxic picoline-borane complex (pic-BH_3_) as reducing agent [16]. In addition, an improved purification process has been developed based on the precipitation of the final product with acetone, the rinsing with acetone to remove water and residual reagents, and the vacuum drying over P_2_O_5_ [17]. These improvements, combined with a re-drawing of the synthetic steps, have therefore made it possible to reduce the time required for obtaining the final product and to scale-up the production of oligosaccharide-derivative of chitosan to the scale of kilograms. As an example, a lactose-derivative of chitosan, indicated as CTL, is now commercially available in medical grade.

### 2.2. Amide Bond Formation

**Scheme 2:**
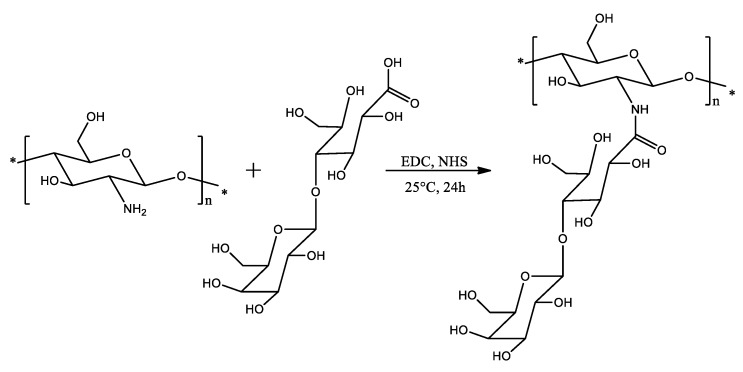
Amide bond formation between chitosan and lactobionic acid mediated by 1-ethyl-3-(3-dimethylaminopropyl)-carbodiimide (EDC) and N-hydroxysuccinimide (NHS). Redrawn from [12,18].

The condensation between a carboxylic group on oligosaccharides and the free amino groups of chitosan is another method exploited to obtain glycosylated-chitosans. The carbodiimide-mediated condensation has been largely employed to easily obtain such soluble derivatives of chitosan. Compared to the approach presented in the previous section, the amide bond formation requires an additional step represented by the chemical modification of the oligosaccharide to introduce the carboxylic moiety. A wide range of products containing a carboxylic acid group have been inserted on the amino groups of chitosan by means of 1-ethyl-3-(3-dimethylaminopropyl)-carbodiimide (EDC) and N-hydroxysuccinimide (NHS) as coupling reagents (Scheme 2) [12,18].

Focusing on saccharide derivatives, lactobionic acid has been largely employed for obtaining biocompatible and bioactive modified chitosans. Indeed, this condensation reaction has been exploited to obtain galactosylated derivatives able to interact with hepatocyte cells [19,20].

### 2.3. Maillard Reaction

**Scheme 3:**
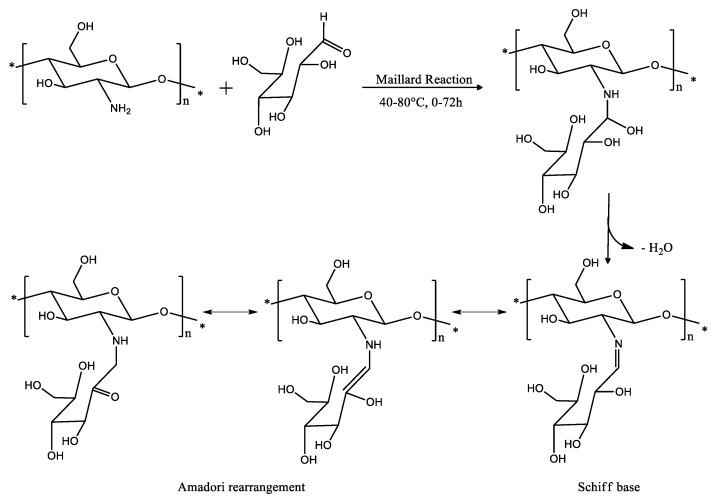
Mechanism of the Maillard reaction. Redrawn from [14].

The Maillard reaction, a form of non-enzymatic browning, is a chemical reaction involving the condensation between an amino group of proteins, amino acids, or any other nitrogenous compound and a carbonyl group of reducing sugars, i.e., aldehydes or ketones [21]. The presence of free amino groups makes chitosan a good candidate to react with reducing sugars through a Maillard reaction (Scheme 3).

Scheme 3 shows that dehydration could follow the Maillard reaction, eventually leading to an Amadori rearrangement. The formation of these compounds can be influenced by many factors, including temperature and time of heating, pH conditions, the concentration and nature of the reducing sugar employed. It has been well reported that the Maillard reaction products contribute important functional properties in foodstuffs [22]. For these reasons, the reaction of chitosan through the Maillard reaction has been largely studied and an impressive characterization has been carried out on a library of products [14,23].

## 3. Physical-Chemical Properties of Oligosaccharide-Derivatives of Chitosan

**Figure 1:**
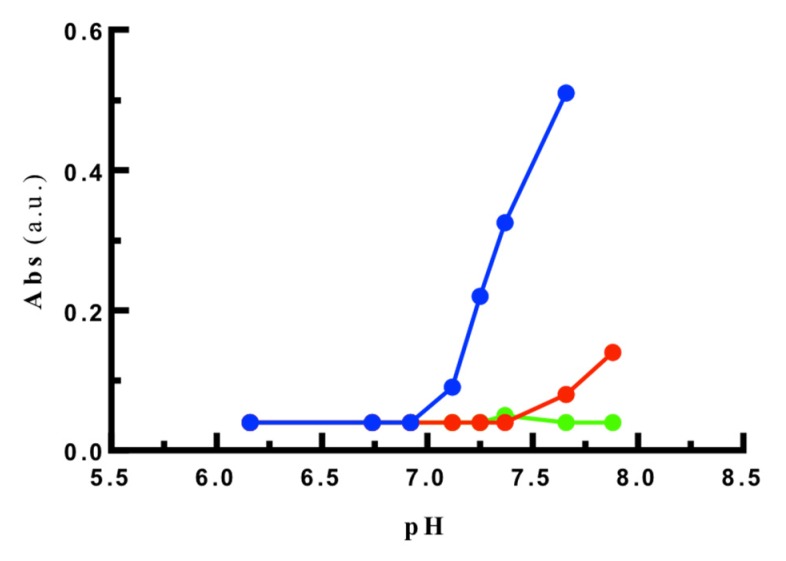
Dependence of the solubility, measured as absorbance, from the pH of the solution for oligomers of chitosan (blue), oligo-substituted chitosan (red), and self-branched chitosan (green). Data redrawn from [26].

The modification of chitosan backbone with oligosaccharide side chains sensibly alters the physical-chemical properties of the product. Among the latter, solubility at neutral pH is considered of paramount importance in devising novel applications for these products. Chitosan is a polycation and its linear charge density decreases upon increasing the pH of the medium. Indeed, the low charge density of chitosan at physiological pH, and the consequent reduction of chain-chain electrostatic repulsion, leads to a low solubility and, hence, to a low stability of chitosan-based formulations. Most of the physical-chemical analyses have been performed on glycosylated chitosan samples arising from reductive *N*-alkylation. The introduction of oligosaccharide side chains has been reported by several authors to circumvent this limitation. Indeed, 40% of oligosaccharides introduced in the chitosan backbone suffices to render the branched polycation soluble at all pH values [24]. Similarly, a complete solubility of the lactose-modified chitosan is found at neutral pH, regardless of the ionic strength used [25]. A more extended analysis performed on short chain polysaccharides has shown that the linear chitosan sample precipitates at pH values higher than 7. At variance, the presence of oligomers, and even more self-branching, increases the complete solubility up to at least pH 8 (Figure 1) [26].

The apparent pKa value of the primary and secondary amino groups of glycosylated chitosan is evaluated by means of ^1^H-NMR titrations from the shift of the proton at C2 position of the chitosan backbone. Equation (1) determines the “apparent” pKa for the two amino groups [27]:

(1) $$pKa_{app}\left(\alpha \right)=pH+log\left(\frac{1-\alpha }{\alpha }\right)$$

where α is the ionization degree. The “apparent” pKa is related to the dissociation constant of the first acid/base group in the uncharged chain as follows (Equation (2)):

(2) $$pKa_{app}\left(\alpha \right)=pKa_{0}+\Delta pKa\left(\alpha \right)$$

where (Equation (3))

(3) $$\Delta pKa\left(\alpha \right)=\frac{1}{n_{p}2.303\;RT}\;\frac{\partial G^{ion}\left(\alpha \right)}{\partial \alpha }$$

with *n_p_* the number of polymeric charge units and *G^ion^* the ionic free energy function [28,29].

The Katchalsky plot at α = 0.5 provides the apparent pKa for the primary and secondary amino groups for the glycosylated chitosan. In the work by Tømmeraas and co-workers [24], the apparent pKa of the primary amino group of chitosan is reported to be 6.9, while the secondary amino group of glycosylated chitosan shows a value of 5.2. Very consistently, the values 6.69 and 5.87 were calculated for the primary and secondary amino groups of a lactose-modified chitosan, respectively [27]. The lower values of pKa for the modified amino functionality with respect to the primary amine is attributed to the possible formation of hydrogen bonds within the polysaccharide structure, which alter the dissociation equilibrium.

The characterization of the shape and overall hydrodynamic compactness for oligosaccharide-branched chitosans can be performed by evaluating the contraction factor, *g* (Equation (4)), which is the ratio between the mean square radius of the branched molecule and the mean square radius of gyration of the linear molecule of the same molecular weight in the unperturbed state.

(4) $$g=\frac{{\left\langle R_{\theta }^{2}\right\rangle }_{br}}{{\left\langle R_{\theta }^{2}\right\rangle }_{lin}}$$

It has been reported that the introduction of branches on the chitosan backbone leads to a more compact structure. As a consequence, the introduction of branches decreases the intrinsic viscosity of the structure [30].

**Figure 2:**
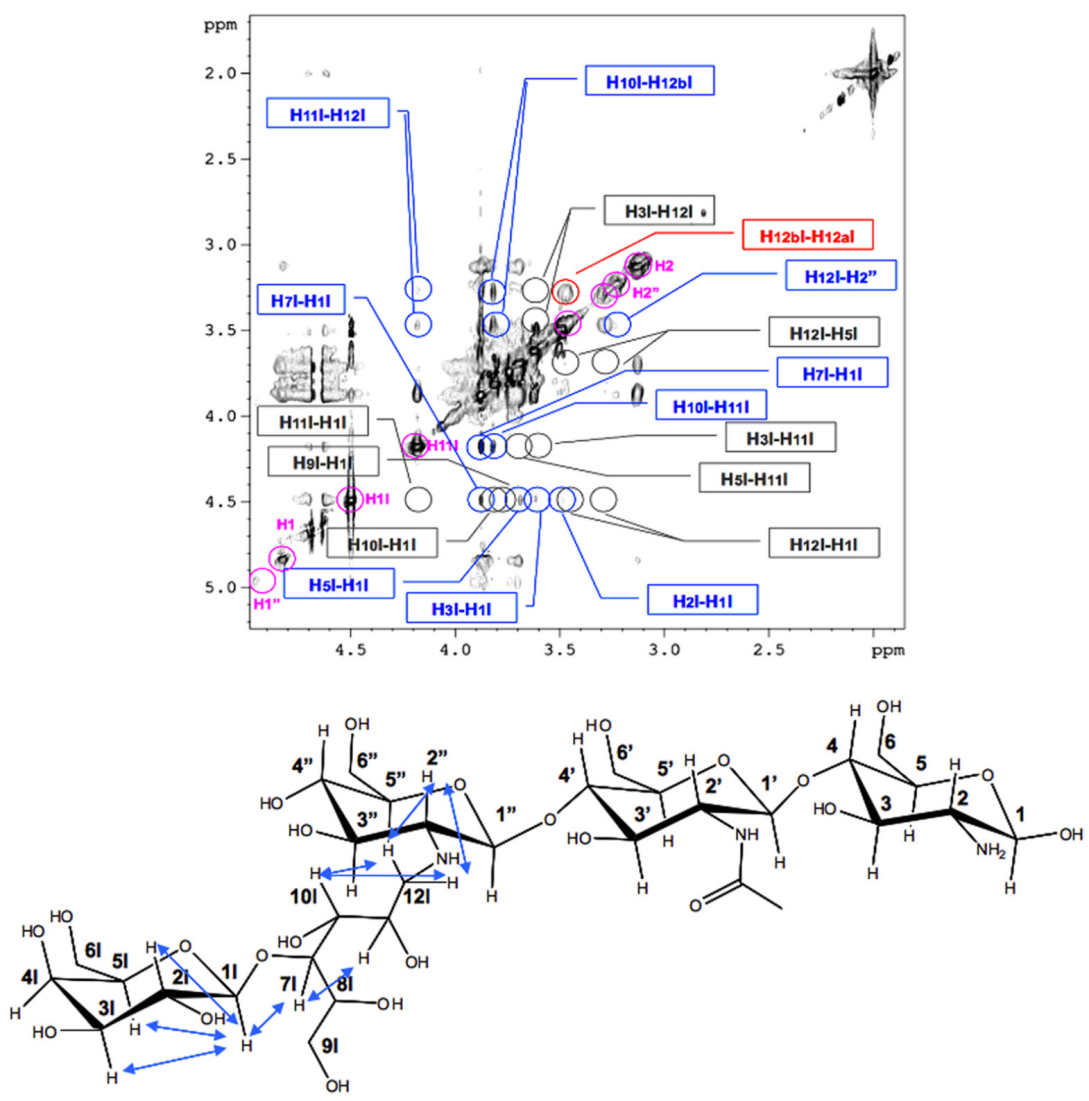
(**Top**) ^1^H, ^1^H-2D NOESY spectrum of lactose-modified chitosan in D_2_O. Unambiguous meaningful NOEs are shown in blue. Empty regions indicating the absence of NOE between two protons are shown in dark gray; the NOE peak used for distance calibration connecting H12 geminal protons (distinguished with letters a and b) are shown in red. Other diagonal peaks are shown in magenta. (**Bottom**) NOE network regarding the lactitol moiety is shown on the polysaccharide structure. Reprinted with permission from *Phys. Chem. B* 2013, 117, 43, 13578–13587. Copyright (2013) American Chemical Society.

NOESY spectra of lactose-modified chitosan reveal that the ring in the side chain adopts a chair conformation with the larger substitute in equatorial position. In addition, relaxation measurements show a substantial degree of mobility for the lactitol moiety [27], and suggest that the orientation of the flexible side chain points away from the chitosan backbone (Figure 2). Although long-range interactions are excluded, NMR data cannot rule out the presence of hydrogen bonds between the glycosylated side chain and the chitosan backbone, as suggested by the lower mobility observed for some protons assigned to the reacted oligosaccharide.

The dynamics of the oligosaccharide side chains in modified chitosan were explored by measuring R_1_ and R_2_ relaxation rates [27]. ^1^H-, ^13^C-HSQC spectra reveal that R_2_ values are visible only for the oligosaccharide side chain, given to the shorter lifetime of the signals assigned to the chitosan backbone due to slow molecular tumbling. The fast motion of the oligosaccharide moiety suggests that it is unlikely to interact with the chitosan backbone and all interactions of the flexible side chains are short-lived and unable to stabilize defined conformations. Overall, the motion of the glycosylated chitosan slows down upon increasing the amount of side chains inserted on the backbone, likely due to the increase of molecular weight, although an effect of stronger interactions with the solvent cannot be ruled out.

**Figure 3:**
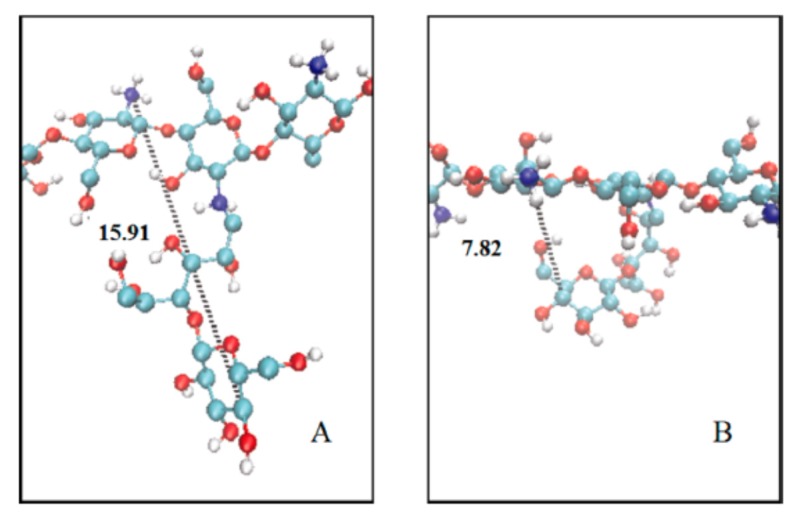
Molecular Dynamics (MD) snapshots showing the orientation of the lactitol side attached to the chitosan backbone in the regions of conformational switches I (**A**) and II (**B**). The distance (in Å) between atom C4l at the far end of lactitol and the N5 atom of the preceding chitosan repeating unit is reported in each case. Reprinted with permission from *Phys. Chem. B* 2013, 117, 43, 13578–13587. Copyright (2013) American Chemical Society.

Molecular Dynamics (MD) calculations show the existence of two conformations of the oligosaccharide chains with respect to the chitosan backbone: an extended one, in which the side chain extends outward with respect to the polysaccharide axis, and a folded one, in which the oligosaccharide side-chain becomes parallel with the chitosan backbone (Figure 3) [27]. The propensity of adopting helix-like local geometries of oligosaccharide-side chains have been also recently studied [31]. At low ionic strength, a 3_2_ helical geometry is mainly attained while the preference is shifted towards a 2_1_ geometry when electrostatic interactions are screened. Both these helical structures for the glycosylated-chitosan samples belong to the same potential well, showing that small changes in polymeric and solution conditions might alternatively favor one of the two helices [31].

**Figure 4:**
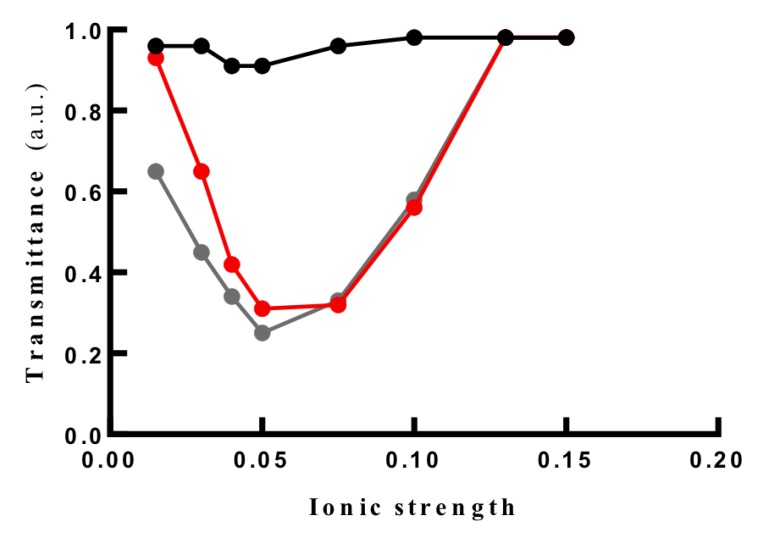
Dependence of the solubility, measured as transmittance, from ionic strength for binary mixtures of alginate and lactose-modified chitosan at a weight ratio of polyanion over polycation of 0.75 (black), 0.25 (red), and 0.15 (grey). Data redrawn from [25].

The presence of glycosylation, besides altering the solubility of the modified polysaccharide at non-acidic pH values, determines an interesting and peculiar spectrum of interactions with polyanions. In fact, the saccharide branching alters the binding of the polycation to DNA. In particular, the efficiency of transfection on HeLa cells increases up to 70% when saccharide branched chitosan is used, while the percentage is almost null when the linear polysaccharide is employed [32]. In addition, the introduction of oligosaccharides on chitosan backbone renders the complexes with DNA more stable in Phosphate Buffered Saline (PBS) and Hanks Balanced Salt Solution [33]. It has been reported that lactose-modified chitosan, when treated with a highly charged polyanionic polysaccharide, such as alginate, allows to obtain, depending on the ionic strength used, a completely non-interacting system, a phase separated polyanion-polycation aggregate, or soluble complexes (Figure 4). The final form of the polycation–polyanion system is also strongly dependent on the ratio between the two charged polymers. Specifically, when a polyanion/polycation ratio from 0.75 to 0.25 is used, the transition from non-interacting polymers to a phase separated system to soluble complexes is attained upon reducing the ionic strength. At variance, when the polyanion/polycation ratio of 0.15 is used, the formation of soluble complexes is not detected [25].

The formation and stability of lactose-modified chitosan (CTL)/hyaluronan (HA)-based complex coacervates was recently investigated [34]. Specifically, CTL and HA typically assembled at acidic pH values due to electrostatic interactions between positive charges on CTL and negative ones on HA, and to the entropy gain following the release of counterions from polyelectrolytes. Colloidal stability may be dramatically altered upon increasing the pH. Specifically, CTL/HA-based coacervates were found to dissolve for pH > 6 due to the loss of positive charges on CTL. However, dissolution stability can be improved by lowering the degree of substitution of glycosylated chitosan from 60% to 47% due to a different balance of primary/secondary amines. Moreover, it was reported that the storage stability is ensured following trehalose addition prior to the freeze-drying process. The formation of soluble complexes containing the oligosaccharide-modified chitosan and highly charged polyanions is of particular interest as synergistic interactions take place, increasing both the viscous and mechanical response of the system, with respect to ones shown by the polymer components considered separately [35].

**Figure 5:**
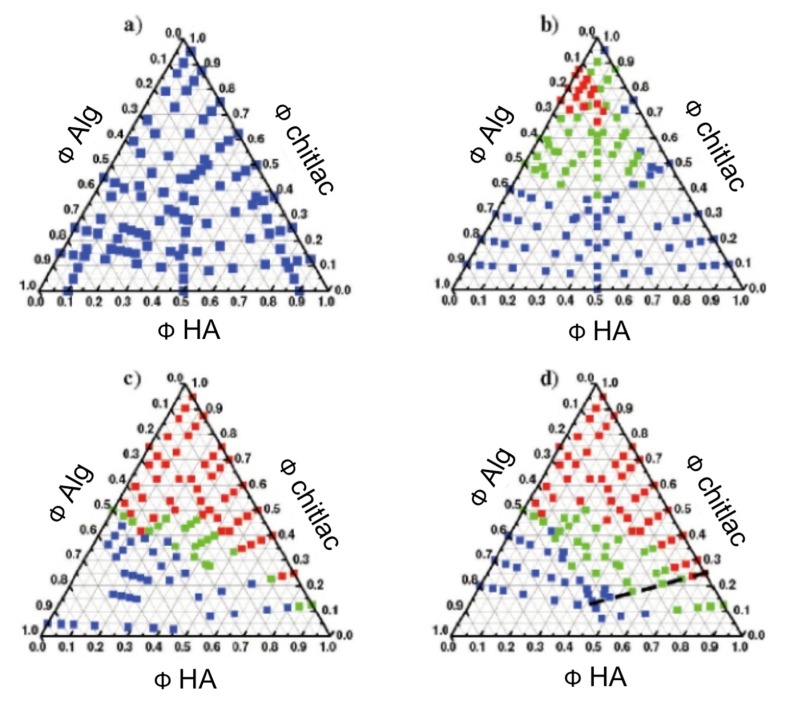
Miscibility of ternary mixtures of alginate (Alg), hyaluronan (HA), and lactose-modified chitosan (chitlac) in aqueous (**a**) NaCl 0.15 M; (**b**) NaCl 0.075 M; (**c**) NaCl 0.015 with a total polymer concentration of 1 g/L, and (**d**) in aqueous NaCl 0.015 M, with a total polymer concentration of 1.5 g/L. φ_Alg_, φ_HA_, and φ_chitlac_ refer to the weight fraction of alginate, hyaluronan, and lactose-modified chitosan, respectively. Ternary compositions corresponding to soluble systems, to phase-separated aggregates, and to soluble complexes are represented in blue, red, and green squares, respectively. Adapted with permission from *Biomacromolecules* 2011, 12, 11, 4044–4056. Copyright (2011) American Chemical Society.

Interestingly enough, oligosaccharide-modified chitosan allows, also, the formation of ternary systems with two polyanions with the formation of a non-interacting system, soluble complexes, and coacervates, depending on the overall ionic strength and on the weight fractions between the three components (Figure 5) [36,37].

## 4. Glycosylated-Chitosan Derivatives for Drug/Gene Delivery 

Given their unique physical-chemical properties, different types of glycosylated-chitosan derivatives have demonstrated great potential as vehicles for drug and gene delivery applications. Popa and co-workers developed polymeric magnetic nanoparticles based on a chitosan-maltose derivative [38]. Nanoparticles were synthetized by joint ionic and covalent crosslinking, and were suitable to encapsulate and release the 5-Fluorouracil antitumor drug. Similarly, Gui and co-workers synthetized a galactose-grafted chitosan and used it to cover the surface of poly(lactic-co-glycolic acid) nanoparticles loaded with TNF-α siRNA [39]. The authors demonstrated improving the RAW 264.7 macrophage-targeting kinetics of nanoparticles in vitro. Furthermore, the presence of the glycosylated chitosan was effective in alleviating in vivo inflammation more efficiently than nanoparticles based on unmodified chitosan.

Similarly, Liu and co-workers developed a galactosylated chitosan and used it to cover carboxyl-modified mesoporous silica nanoparticles. The nano-drug delivery system was assembled to load calcium leucovorin molecule, and was found to target, selectively, colon cancer cells showing high expression of galectin receptor [40]. Qi et al. described the synthesis of galactosylated chitosan-grafted oxidized carbon nanotubes for the pH-dependent sustained release and hepatic tumor-targeted delivery of doxorubicin [41]. Overall, these nano-carriers manifested potent in vitro tumor-targeting properties, with cellular uptake efficiency higher than that of free doxorubicin in HepG2 cells. Moreover, they proved, in vivo, higher antitumor activity compared to free doxorubicin.

Webster and co-workers recently designed galactosylated chitosan-based nanoparticles with high drug loading capacities for targeted delivery toward hepatocellular carcinoma [20]. This drug delivery system exhibited superior efficacy while mitigating systemic toxicity. Furthermore, it induced cancer cell apoptosis via blocking TNF/NF-κB/BCL2 signaling. Taken together, these findings provide evidence that this drug delivery system might be a potential therapeutic agent against hepatocellular carcinoma.

Wang et al. developed a novel drug delivery system comprising nanoparticles based on a galactosylated chitosan/graphene oxide/doxorubicin envisaged for the treatment of cancer [42]. In vitro experiments demonstrated that this type of drug delivery system showed higher cytotoxicity for cells than pure chitosan/graphene oxide/doxorubicin system. Furthermore, anti-tumor experiments proved that the nanoparticles constituted of galactosylated chitosan inhibited tumors in vivo. Lactose-modified chitosan was also used in combination with hyaluronic acid to produce nanogels for tumor targeting and controlled delivery of doxorubicin and nitric oxide. The release of the two drug targets was initiated by the cleavage of cyclic boronate esters [43]. Coya et al. synthetized a tri-mannose derivative of chitosan for coating nano-carriers and study their impact on human macrophages response. It was strikingly found that functionalized particles affected cell metabolism in terms of oxidative phosphorylation and sugar metabolism; this led to consider such system for reprogramming immune cells and improving the efficacy of encapsulated drugs [44].

**Figure 6:**
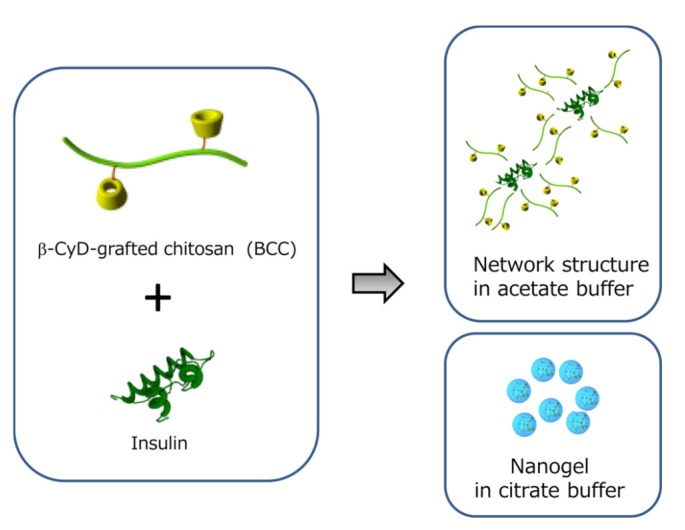
β-cyclodextrin-grafted chitosan based complexes able to suppress glucose levels. Reprinted with permission from *Molecular Pharmaceutics* 2016, 13, 12, 4034–4042. Copyright (2016) American Chemical Society.

Ariga and collaborators produced a β-cyclodextrin-grafted chitosan, assembled complexes in the presence of insulin, and investigated the effect of carrier morphology on the intestinal absorption of insulin (Figure 6) [45]. Following to oral administration in mice, the authors demonstrated the effective suppression of glucose levels. This carrier has, therefore, great potential as an absorption enhancer for peptide/protein drugs.

**Figure 7:**
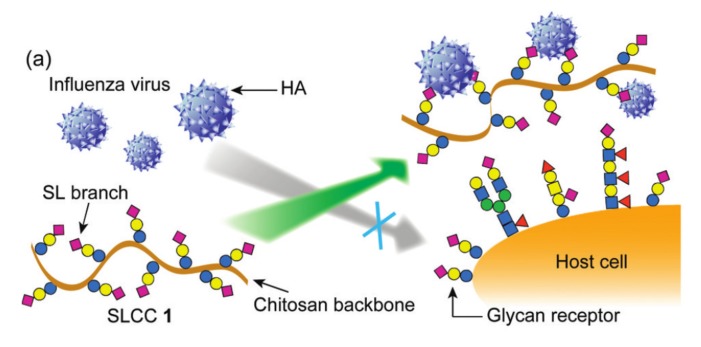
Sialyllactose-chitosan derivative as potential virus adsorbent for prevention and control of influenza. Adapted with permission from *Biomacromolecules* 2011, 12, 11, 3962–3969. Copyright (2011) American Chemical Society.

Li et al. synthetized a sialyllactose-chitosan derivative by grafting a lactoside bearing an aldehyde-functionalized aglycone to the amino groups of chitosan, followed by the enzymatic sialylation with sialyltransferase (Figure 7). Resulting glycosylate chitosan was found particularly appealing in binding the influenza virus surface hemagglutinin protein with high affinity; thereby, inhibiting the viral attachment to host erythrocytes [46]. Authors concluded that this chitosan conjugate may function as a potential virus adsorbent for prevention and control of influenza.

Vårum and co-workers synthetized a chitosan derivative substituted with sugar oligomers, in which depolymerized chitosans were reductively *N*-alkylated with the trimer A-A-M. This glycosylated chitosan was employed as gene delivery system in forming polyplexes in the presence of DNA or small interfering RNA (siRNA) [47,48]. Specifically, it was shown that the most efficient gene silencing in mammalian cells was achieved using fully de-*N*-acetylated chitosans with intermediate chain lengths causing minimal cytotoxicity.

Chitosan was modified also by fructose and used to form self-assembled nanoparticles loading doxorubicin as a model anticancer drug [49]. Yin et al. synthetized a glucosyloxyethyl acrylated chitosan suitable for forming genipin-crosslinked glucose-responsive microgels [50]. The microgels were prepared through a reversed-phase emulsion crosslinking method. This microgel could be a promising system for self-regulating insulin delivery, as well as other applications, such as actuators and separation systems with sensitivity to glucose. Sacco et al. used a lactose-derivative of chitosan for the formation of simple or complex coacervates in the presence of tripolyphosphate as multivalent anion, and negatively charged macromolecules, such as hyaluronan [34,51]. These nanosystems were highly monodisperse and efficiently hosted payloads. In vitro experiments towards human neutrophils demonstrated a potent radical scavenger activity. Given the well-documented bioactivity of this engineered polymer, these findings suggest a possible application of the glycosylated chitosan-based drug delivery system as scavenger and bioactive carrier for drug therapeutics at confined inflamed sites. Furthermore, the lactose-derivative of chitosan has been recently employed in forming gold-based nanoparticles with potential translation of present systems in both treatments of tumors and glucose sensor [52,53].

## 5. Glycosylated-Chitosan Derivatives for Tissue Engineering 

Chitosan and its derivatives are widely used in the tissue-engineering field due to their structural similarity to the glycosaminoglycans of the extracellular matrix (ECM). Introducing cell-specific ligands or extracellular signaling molecules on a polymer array represents a good strategy for tissue regeneration. In the field of liver regeneration, chitosan modified with galactose moieties is advantageous for the in vitro growth of hepatocytes. Indeed, the proliferation of hepatocytes requires a matrix that not only mimics ECM components, but also allows for the binding between scaffolds and the cell surface receptors [54]. The use of galactosylated chitosan as synthetic ECM for liver tissue engineering and cell adhesion, spreading, and proliferation of hepatocytes was described by Park et al. [55] The ability of hepatocytes to proliferate on this matrix and to form spheroids is due to the fact that the asialoglycoprotein receptor (ASGP-R) is overexpressed on their surface and selectively binds to galactose. Previous studies have already shown improvements on the viability and spheroid formation by hepatocytes on scaffold upon addition of galactosylated chitosan.

In particular, Chung et al. fabricated highly porous, three-dimensional scaffolds by reacting galactosylated chitosan with Ca^2+^-alginate gel through electrostatic interactions between the carboxylic groups of alginate and the amino groups of chitosan derivative [18]. Galactosylated chitosan was used in this work, not only to improve the attachment of hepatocytes on the scaffold, but also to ameliorate the mechanical properties of alginate. Indeed, scanning electron microscopy analyses revealed that the porosity and pore size of the sponge were highly dependent on the content and molecular weight of chitosan derivative and on the freezing temperature. Furthermore, the improvement in mechanical properties correlated with the amount of glycosylated chitosan.

Chen and co-authors suggested another strategy to fabricate scaffold based on galactosylated chitosan and alginate. They reported a scaffold for liver tissue engineering composed of oxidized alginate covalently cross-linked with galactosylated chitosan via the Schiff’s base reaction, without employing any additional crosslinking agent. In vitro tests suggested a good biocompatibility and a favorable biological response of hepatocytes to the treatment with the material [56]. Another improvement on scaffold for liver tissue engineering was reported by Seo et al. [57]. They suggested a long-term co-culture of hepatocytes with NIH-3T3 cells on a porous hydrogel scaffold based on alginate, galactosylated chitosan, and heparin. The components were joined together by electrostatic interactions. The addition of heparin was due to its high affinity for hepatocytes growth factor (HGF), which is well known as a potent mitogen, mitogen, and morphogen agent involved in growth regulation of hepatocytes. Hybrid sponges for co-culture were also obtained by mixing galactosylated chitosan and hyaluronic acid. Shang et al. fabricated highly porous galactosylated chitosan-based sponges that provided an ECM-like environment suitable for a co-culture of hepatocytes and endothelial cells. It is reported that hepatocytes interacted with sponge through the ASGP-R, while endothelial cells used the CD44 receptor [58].

In order to mimic the spatial architecture of extracellular matrix, nanofibrous structures have been investigated over the last few years. Feng et al. reported galactosylated chitosan-based nanofibrous scaffolds with uniform distribution of fiber diameters [59]. The interaction between this type of scaffold and primary hepatocytes was evaluated in terms of cell adhesion, morphology, packing, bioactivity, and liver-specific function maintenance. The results suggest that galactosylated chitosan nanofibrous scaffolds could be used as hepatocyte culture substrates. Kasoju and Bora suggested the use of electrospinning to obtain nanofibrous scaffolds made of silk fibroin and galactosylated chitosan [60]. Poly(ethylene oxide, PEO) was also added in order to obtain nanofibers with uniform size distribution. Furthermore, in vitro tests have been performed to investigate on hepatocytes homeostasis on this type of substrates. Contextually, Qui et al. discussed a preparation of mixed scaffolds made of galactosylated chitosan and polycaprolactone (PCL) [61]. In this paper, PCL scaffolds—prepared by a method of gelatin particle leaching—were hydrolyzed to produce carboxylic groups that were employed to react with the amino groups of the galactosylated chitosan. Thanks to this modification, the resulting scaffolds could be better recognized by hepatocytes and showed an improved cell viability, spheroids formation, and long-term maintenance of liver-specific function, such as albumin secretion.

The use of galactosylated chitosan is not the only solution available to improve hepatocytes attachment and maintenance of cell viability. Another suitable specific ligand for cell receptors is fructose. For instance, Evdokimova et al. showed a protective behavior of this monosaccharide on hepatocytes [62]. Li et al. conjugated fructose onto the inner surface of highly porous chitosan scaffold assembled by freeze-drying [63]. As a result, they obtained scaffolds promoting improved cell metabolic activity, in terms of albumin secretion and urea synthesis by hepatocytes, with respect to cells atop unmodified sponge counterparts.

Lactose-modified chitosans are often cited for their applications in different fields, such as liver, bone, cartilage, and nerve tissue engineering. Wang and co-workers synthetized a lactose-modified chitosan with a various degree of substitution for hepatocytes growth [64]. The chemical modification of chitosan with lactose seems to be a good choice, not only for the liver, but also for cartilage tissue engineering [65]. In particular, modified chitosan with lactose and heparin can improve chondrocytes growth in vitro. This substrate showed stronger ability to induce chondrocytes attachment, proliferation, viability, and glycosaminoglycans secretion than simple chitosan films. Indeed, heparin is known to bind a number of biologically important proteins as well as growth factors; thus, the chitosan derivative combined with heparin can create a biomimetic microenvironment [66,67,68,69]. The same research group suggested another strategy to use lactose-grafted chitosan for cartilage regeneration purposes [70]. In this work, they used poly(l-lactide) (PLLA) microspheres that were modified in order to couple, via Schiff’s base formation, a lactose-modified chitosan. In this way, PLLA would act as a microcarrier that supports cell attachment and induces aggregation on the surface. These aggregates showed higher viability and extracellular matrix production. For these reasons, microcarriers can be potentially used as an injectable delivery system for cartilage repair.

Donati et al. described, in 2005, a lactose-modified chitosan named Chitlac (now commercially indicated as CTL) [15]. This polymer displays intriguing biological properties that have been related to its interaction with Galectin-1 [71]. In vitro studies performed on primary chondrocytes pointed out that CTL induced cell aggregation and proliferation, and stimulated the synthesis of collagen and GAGs. The polymer CTL has been grafted on the surface of the BisGMA/TEGDMA thermoset employed for dental and orthopedic applications, in order to confer bioactive properties to these materials [72]. Material functionalization is based on the acidic treatment of the thermoset surface, leading to the exposure of carboxyl groups; the latter can then establish electrostatic interactions with the bioactive polymer CTL, which confers to materials, physical-chemical properties that are close to those of the hydrated extracellular matrix. CTL-based constructs have been also employed for applications in the regeneration of central nervous systems. The results showed that CTL-based substrates can promote neuronal growth and differentiation, together with the formation of new synapses [73].

The polymer CTL has been studied, also, for its ability to form polysaccharide-based matrices in which silver nanoparticles (nAG) can be embedded. Silver nanoparticles display antimicrobial activity towards Gram^+^ and Gram^−^ bacteria strains, which make them good candidates for the development of biomaterials endowed with antimicrobial properties [74,75]. Despite this biological activity, when used as such, they display a poor biocompatibility on eukaryotic cells. However, the entrapment into a polysaccharidic matrix prevent nAG particles to be uptaken by cells. It has been reported that CTL-nAG complexes can exert their antimicrobial activity by interacting with thiol groups of bacterial membranes; thus, leading to cell death. At variance, the presence of the polysaccharidic matrix-embedding nAG prevents the internalization of the nanoparticles by cells, which results in increased biocompatibility. The biological properties of CTL-nAG complexes have been exploited for the development of BisGMA/TEGDMA coated with CTL-nAG, in order to develop biomaterials endowed with antimicrobial properties and biocompatibility on cells [76].

One of the main advantages related to the development of these coated-biomaterials refers to the possibility of killing bacteria by a direct interaction with the material surface, without the release of components that can be harmful to cells and threaten biocompatibility. The material was prepared by a chemical process that led to the exposure of carboxylic groups on the surface of the thermoset, which was then immersed in a CTL-nAG solution in order to enable the formation of electrostatic interactions between the activated COO^-^ groups and the positively charged molecules of the polysaccharide. The results showed that the CTL-nAG coating was effective in exerting its antimicrobial activity towards both Gram^−^ and Gram^+^ bacteria, while the biocompatibility on cells was preserved. The eukaryotic cells cultured on the surface of the coated thermoset were able to adhere and proliferate on material surface, which supports the use of such materials for the development of biomaterials and medical devices in different fields, such as orthopedics and dentistry [76]. A further characterization of these CTL-nAG coated BisGMA/TEGDMA thermoset showed that the coated material displayed anti-biofilm properties by inhibiting bacterial adhesion and proliferation. An in vivo characterization was performed by implanting these biomaterials in the femur of minipigs, in comparison with a Ti6Al4V alloy material. The histological results showed the biocompatibility of the implants and the absence of significant differences in bone response among the tested implants [77].

Polymeric solutions containing CTL can form dynamic polymeric networks in the presence of inorganic crosslinkers, such as boric acid, which can bind flanking diols [78,79]. These hydrogel systems display peculiar mechanical properties, since a strain-hardening behavior can be observed when a mechanical stress is applied on them. This mechanical response is characterized by a linear response to applied stress, followed by an exponential increase of the elastic modulus of hydrogels upon exceeding a critical strain. This behavior mimics that of some components of the extracellular matrix of cells and appears fundamental to carry out some biological functions [80]. Further developments of CTL-boric acid hydrogels were based on the possibility to increase the homogeneity of hydrogels and to control the gelation process [81]. In these works, the authors showed that over a certain polymer-crosslinker ratio, the addition of the inorganic agent leads to the instantaneous formation of inhomogeneous aggregates. At variance, in the presence of competitors of the polymer CTL, such as mannitol, the crosslinker can associate with the latter and can further form complexes with the CTL polymer, leading to the formation of homogeneous hydrogels [82]. In another work, the use of sodium bicarbonate (NaHCO_3_) has been proposed to control the gelation process. Indeed, the interaction of CTL with boric acid is dependent on pH. In the proposed system, the two components are mixed at acid pH (pH = 5) at which the reactivity of boric acid is low. The addition of NaHCO_3_ leads to a slow increase of pH, which allows the conversion of boric acid into a more reactive form (the borate anion), which favors the formation of borate esters with CTL diols [81]. Table 1 summarizes glycosylated chitosans envisaged for Tissue Engineering applications.

## 6. Outlook

Chitosan is regarded as an interesting and promising polysaccharide for several advanced applications ranging from drug delivery to biomaterials design. However, physical-chemical characteristics, such as low solubility at neutral pH, hamper its wide use. In this perspective, glycosylation appears as a very promising approach to upgrade the parent polysaccharide along two directions at the same time: increase of the solubility and targeting towards specific receptors. Some of the advantages of glycosylated chitosans are at present being explored, although it is conceivable that their potential has yet to completely emerge. Indeed, the combination of an increasing knowledge in glycobiology, role of glyco-signals and a complete understanding of their physical-chemistry can render these polymeric molecules of interest for advanced medical applications. In this sense, glycosylation could represent an innovative and chemically simple approach to widen chitosan applications and, thus, could contribute to the use of this renewable resource from natural origin in several application fields.

## Figures and Tables

**Table 1:** Resume of glycosylated chitosans and related Tissue Engineering applications.

Glycosylated Chitosan	Applications	Refs.
Galactosylated chitosan	Synthetic Extracellular Matrix for liver regeneration (in vitro)	Park et al. [55]
Galactosylated chitosan and alginate	Scaffolds for liver tissue engineering	Chung et al. [18] Chen et al. [56] Seo et al. [57]
Galactosylated chitosan and hyaluronic acid	Sponge for liver tissue engineering	Shang et al. [58]
Galactosylated chitosan	Nanofibrous scaffolds for liver tissue engineering	Feng et al. [59]
Galactosylated chitosan and silk fibroin	Nanofibrous scaffolds for liver tissue engineering	Kasoju & Bora [60]
Galactosylated chitosan and polycaprolactone	Scaffolds for liver tissue engineering	Qiu et al. [61]
Fructose-modified chitosan	Scaffolds for liver tissue engineering	Li et al. [63]
Lactose-modified chitosan and poly(l-lactide)	Microspheres for cartilage tissue engineering	Tan et al. [70]
Lactose-modified chitosan	Substrates for liver regeneration	Wang et al. [64]
Lactose-modified chitosan	Substrates for cartilage regeneration (in vitro)	Tan et al. [65]
Lactose-modified chitosan	Thermoset materials for dental and orthopedic applications	Travan et al. [72]
Lactose-modified chitosan	Constructs for the regeneration of central nervous system	Medelin et al. [73]
Lactose-modified chitosan and silver nanoparticles (nAG)	Materials based on glycosylated chitosan and silver nanoparticles endowed with antimicrobial properties for wound, orthopedic, and dental applications	Travan et al. [74] Sacco et al. [75] Travan et al. [76] Marsich et al. [77]
